# Berberine suppresses gero-conversion from cell cycle arrest to senescence

**DOI:** 10.18632/aging.100593

**Published:** 2013-08-21

**Authors:** Hong Zhao, H Dorota Halicka, Jiangwei Li, Zbigniew Darzynkiewicz

**Affiliations:** Brander Cancer Research Institute and Department of Pathology, New York Medical College, Valhalla, NY 10595, USA

**Keywords:** Berberine, Cellular senescence, H2AX phosphorylation, ROS, ribosomal protein S6, calorie restriction, metformin, rapamycin, 2-deoxyglucose, replication stress, cell cycle

## Abstract

Berberine (BRB), a natural alkaloid, has a long history of medicinal use in both Ayurvedic and old Chinese medicine. Recently, available as a dietary supplement, Berberine is reported to have application in treatment of variety diseases. Previously we observed that BRB inhibited mTOR/S6 signaling concurrently with reduction of the level of endogenous oxidants and constitutive DNA damage response. We currently tested whether Berberine can affect premature, stress-induced cellular senescence caused by mitoxantrone. The depth of senescence was quantitatively measured by morphometric parameters, senescence-associated β-galactosidase, induction of p21^WAF1^, replication stress (γH2AX expression), and mTOR signaling; the latter revealed by ribosomal S6 protein (rpS6) phosphorylation. All these markers of senescence were distinctly diminished, in a concentration-dependent manner, by Berberine. In view of the evidence that BRB localizes in mitochondria, inhibits respiratory electron chain and activates AMPK, the observed attenuation of the replication stress-induced cellular senescence most likely is mediated by AMPK that leads to inhibition of mTOR signaling. In support of this mechanism is the observation that rhodamine123, the cationic probe targeting mitochondrial electron chain, also suppressed rpS6 phosphorylation. The present findings reveal that: (a) in cells induced to senescence BRB exhibits gero-suppressive properties by means of mTOR/S6 inhibition; (b) in parallel, BRB reduces the level of constitutive DNA damage response, previously shown to report oxidative DNA damage by endogenous ROS; (c) there appears to a causal linkage between the (a) and (b) activities; (d) the *in vitro* model of premature stress-induced senescence can be used to assess effectiveness of potential gero-suppressive agents targeting mTOR/S6 and ROS signaling; (e) since most of the reported beneficial effects of BRB are in age-relate diseases, it is likely that gero-suppression is the primary activity of this traditional medicine.

## INTRODUCTION

Cellular senescence can be categorized in two groups. The replicative senescence, seen after certain rounds of cell division in cultures (“Hayflick's limit”) [1], is a consequence of a progressive erosion of telomeres at each division which leads to a telomere dysfunction and irreversible cell cycle arrest [2]. The second category defined as premature cellular senescence is unrelated to telomere shortening [review, 3]. Persistent cellular stress including replicative stress caused by oxidative DNA damage [4,5], activation of oncogenes [6] and loss of tumor suppressor genes [7] are among mechanisms inducing premature senescence. While in certain malignancies, particularly in acute leukemia, chemo- or radio- therapy induces apoptosis, the mechanism of elimination of cancer cells in some solid tumors often relies on irreversible impairment of cell reproductive capability defined as a drug- or radiation-induced senescence, that also belongs to the category of premature senescence [8,9]. Likewise, premature senescence of induced pluripotent stem cells (iPSCs) is a barrier in tumor development [10]. The stress-induced premature senescence of normal cells *in vivo* is considered to be a critical mechanism affecting organismal aging and longevity [11-14].

Extensive attempts have been made to develop gero-suppressive modalities that can slow down processes of senescence and aging extending longevity. Assessment of their effectiveness by analysis of animals' life span, especially when it involves vertebrates [15,16], is cumbersome and time consuming. It is therefore desirable to have relatively rapid *in vitro* approach that can be used for this purpose. Cumulative DNA damage caused by reactive oxygen species (ROS) produced during oxidative phosphorylation for long time was thought to be the major factor promoting aging (ROS mechanism) [17-19]. More recently, however, the persistent stimulation of the mitogen- and nutrient-sensing pathways including mammalian target of rapamycin (mTOR) signaling mechanism has been advanced as an alternative to ROS mechanism [20-28]. Activation of these pathways enhances translation and leads to cell growth in size/mass resulting in cell hypertrophy and senescence. Activation of mTOR/S6K pathway when combined with oxidative DNA damage that leads to replication stress appears to be particularly effective factor promoting aging and senescence [29].

The background level of constitutive activation of ATM and expression of γH2AX seen in untreated normal or cancer cells reports the ongoing DNA oxidative damage and replication stress induced by endogenous ROS [30-32]. Using flow- and laser scanning- cytometry as major methodologies we have recently shown that several reported gero-suppressive agents, namely, rapamycin, metformin, berberine (BRB), 1,25-dihydroxyvitamin D3, the calorie-restriction mimetic 2-deoxyglucose, and acetylsalicylic acid (ASA; aspirin), all depressed the level of constitutive DNA damage signaling [33-35]. Specifically, these substances reduced expression of γH2AX and activation of ATM in a variety of cell types, including tumor A549 and TK6 cells, as well as normal WI-38 cells or mitogenically stimulated human lymphocytes [33]. These agents also decreased the level of intracellular ROS and mitochondrial trans-membrane potential ΔΨm, the marker of mitochondrial energizing [33-35]. The above observations would be consistent with the ROS mechanism of aging. However, all these agents also distinctly reduced the constitutive level of phosphorylation of Ser235/236 of ribosomal S6 protein (rpS6), Ser2448 of mTOR and Ser65 of 4EBP1 [33], the major elements of the mTOR signaling [27,36-38]. Collectively, these data indicated that the reduction of mTOR/S6K signaling, that in turn reduces the translation rate, was coupled with a decrease in oxidative phosphorylation (revealed by ΔΨm) that led to reduction of ROS and attenuated oxidative DNA damage [33]. Thus, while the decreased rate of translation induced by these agents may slow down cells hypertrophy and alleviate other features of cell aging/senescence reduction of oxidative DNA damage may lower predisposition to neoplastic transformation. The latter may result from damage to DNA sites coding for oncogenes or tumor suppressor genes. Our data suggested that combined assessment of constitutive γH2AX expression, mitochondrial activity (ROS, ΔΨm) and mTOR signaling by cytometry can provide an adequate gamut of cell responses to evaluate effectiveness of potential gero-suppressive agents [33].

In continuation of these studies we attempted to explore whether gero-suppressive agents can also attenuate the level of premature, stress-induced cellular senescence. Toward this end we initiated experiments designed to reveal possible effects of these agents on induction of cellular senescence upon exposure of A549 cells to very low concentration of the DNA damaging drugs, shown by us before to trigger DNA replication stress manifesting by ATM activation and induction of γH2AX, that leads to senescence [39,40]. In preliminary experiments we observed that one of the gero-suppressive agents, the isoquinoline alkaloid berberine (BRB), was the most effective, suppressing the induction of cellular senescence at its low, clinically relevant, concentration. The present study, therefore, was designed to explore this effect of BRB in more detail and at the same time to demonstrate utility of flow- and laser scanning- cytometry in multiparametric analysis [41] of the depth of cellular senescence and its modulation by this alkaloid.

## RESULTS

**Figure 1:**
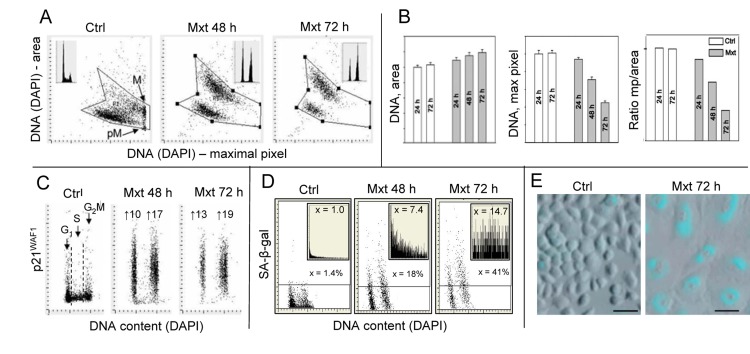
Induction of premature cellular senescence of A549 cells measured by laser scanning cytometry Human pulmonary non-small cell lung carcinoma A549 were untreated (Ctrl) or treated with 2 nM DNA topoisomerase II inhibitor mitoxantrone (Mxt) for 48 or 72 h. Panel **A** shows morphometric features of the cells revealed by measurement of nuclear DNA (DAPI) fluorescence reporting on the bivariate distributions (scatterplots) nuclear area versus intensity of maximal pixel of fluorescence, respectively. Intensity of maximal pixel is correlated with chromatin condensation and in the untreated cells has the highest value and marks mitotic (M) and immediately post-mitotic (pM) G1 cells, which also have low value of DAPI area [41]. In the senescing cells, while nuclear area increases, the intensity of maximal pixel decreases [39,40,64]. These morphometric changes reflect enlargement of the projected nuclear area and decreased DAPI local staining per unit area, due to “flattened” cellular appearance, the hallmark of cellular senescence [42,43]. The insets show DNA content frequency histograms of cells from the respective cultures. Panel **B**: Bar plots reporting mean values (+SD) of nuclear (DNA, DAPI) area, DNA (DAPI) maximal pixel, and ratio of maximal pixel to nuclear area, respectively, of cells from control and Mxt treated cultures. Panel **C**: Bivariate distributions (DNA content vs p21) reporting expression of p21WAF1 with respect to the cell cycle phase; the figures show the n-fold increase in mean expression of p21 of G1 and G2M cells from the Mxt-treated cultures with respect to respective cells in Ctrl. Panel **D**: Bivariate distributions of DNA content versus senescence-associated galactosidase (SA-β-gal) activity. Figures indicate percent of SA-β-gal positive (above the threshold marked by the horizontal lines) cells. Insets show the frequency distribution of SA-β-gal positive cells; the figures in insets show the n-fold increase in the mean activity of SA-β-gal in Mxt-treated cultures over Ctrl (1.0). Panel **E**: Images of cells growing in the absence (left) and presence of 2 nM Mxt for 72 h (right) stained to detect activation of SA-β-gal activity recorded by laser scanning cytometer (Research Imaging Cytometer iCys); 50 μm bars mark the length scale.

One of the most characteristic features of cells undergoing senescence is change in their morphology revealed by an increase in cellular and nuclear size. In the case of cells growing attached this manifests by their dramatic “flattening” appearance combined with markedly reduced cell density at confluence [42,43]. The morphometric analysis of cell nucleus by laser scanning cytometry (LSC) is a sensitive detector of such a change [39,40]. Premature senescence of A549 cells was induced by their exposure to 2 nM DNA topoisomerase inhibitor mitoxantrone (Mxt), the drug that interacts with DNA by intercalation and is a type II DNA topoisomerase inhibitor [44]. Fig. 1 illustrates the features of these cells undergoing senescence, including their morphological changes that were measured by LSC. The morphometric analysis of the nucleus stained with the DNA fluorochrome DAPI shows an increase in nuclear area concomitant with the decrease in intensity of DAPI maximal pixel fluorescence [39]. The integral of intensity of DAPI fluorescence over the nucleus reports DNA content and thus the cell cycle phase, which as seen Fig. 1 (insets), indicates cell arrest in G_1_ and G_2_M with paucity of S-phase cells in the Mxt-treated cultures. Since nuclear area in senescent cells is increased and DAPI maximal pixel decreased the ratio of maximal pixel to the area (Mp:area) provides an even more sensitive marker reporting this change in cell morphology than either of these measurements alone (Fig. 1B). The cells arrested in G_1_ and G_2_M in the Mxt-treated cultures show 10 to 19-fold increase in expression of the CDK inhibitor p21^WAF1^, the another marker of cellular senescence [42,43]. Activation of the senescense-associated β-galactosidase A-β-gal), another hallmark of cellular senescence [42,43] is also markedly elevated in cells growing in the presence of Mxt. The rise in SA-β-gal activity measured by LSC is expressed either as percent of the enzyme-positive cells, which is augmented from 1.4 to 23% and 44%, or the mean value of the light absorption of the SA-β-gal product, increased 8.4- and 17.7-fold, in cells from cultures growing with Mxt for 48 and 72 h respectively (Fig. 1; D,E).

**Figure 2:**
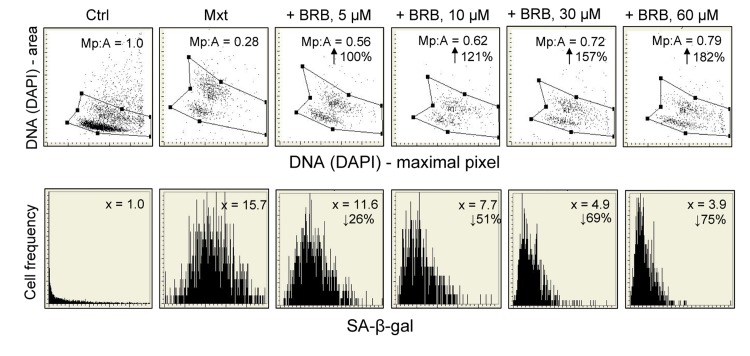
Attenuation of Mxt-induced senescence of A549 cells by berberine (BRB) as measured by cell morphometric features and SA-β-gal activity Exponentially growing A549 cells were untreated (Ctrl) or treated with 2 nm Mxt in the absence and presence of BRB at concentration as shown, for 5 days. Top panels: Morphometric analysis, reporting changes in nuclear area (DNA-DAPI) versus maximal pixel of DAPI fluorescence. The ratio of maximal pixel to nuclear area (Mp:A) is expressed as a fraction of that of the untreated cells; shown is the percent increase in Mp:A in the BRB-treated cultures with respect to cells growing with Mxt alone, with the arrows. Bottom panels: The frequency histograms reporting SA-β-gal activity. The figures present the increase (n-fold) in the enzyme activity with respect to the Ctrl (1.0), measured as the mean intensity of SA-β-gal absorption of cells in the respective cultures.

Induction of senescence of A549 cells growing in the presence of Mxt is distinctly suppressed, in a concentration-dependent fashion, by BRB (Fig. 2). Thus, the morphometric parameter reporting the nuclear change (Mp:area) which in the presence of Mxt alone is decreased from 1.0 to 0.28, in cultures treated with Mxt and 5 μM BRB is decreased to only 0.56, which is a 100% reduction compared to Mxt alone. At higher BRB concentration this effect is more pronounced, and at 60 μM BRB the reduction is 182% vis-à-vis Mxt alone. The attenuation of the Mxt-induced senescence by BRB, when measured by the induction of SA-β-gal activity, is also quite evident, and BRB-concentration dependent. The BRB-induced decrease of SA-β-gal activity in Mxt-treated cells was 26% at 5 μM BRB and further decreased to 75% at 60 μM concentration (Fig. 2).

**Figure 3:**
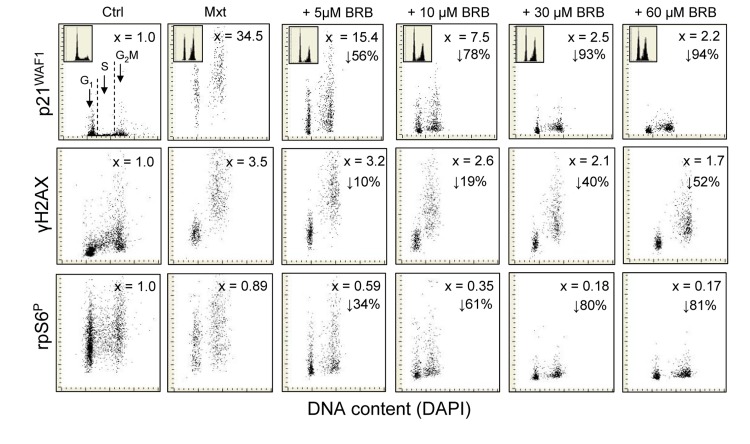
Attenuation of Mxt-induced senescence of A549 cells by BRB as measured by reduction in expression of p21^WAF1^, γH2AX and rpS6^P^ Exponentially growing A549 cells were untreated (Ctrl) or treated with Mxt in the absence and presence of BRB at concentrations as shown, for 5 days. Top panels: bivariate distributions of p21 versus cellular DNA content; the figures (x) present the increase (n-fold) in the mean expression for all cells of p21 with respect to the untreated cells, the percent reduction in p21 in cultures with BRB with respect to Mxt alone, is shown with the arrows. Mid-panels: expression of γH2AX versus DNA content; the figures (x) represent the increase (n-fold) in the mean expression of γH2AX with respect to untreated cells (1.0); the percent reduction in expression of γH2AX in cultures grown with BRB with respect to cells growing in the presence of Mxt alone is presented with the arrows. Bottom panels: expression of rpS6^P^ versus DNA content. The figures illustrate the change (n-fold) with respect to the untreated cells; the percent reduction in expression of rpS6^P^ in cultures with BRB with respect to cells treated with Mxt alone is shown with the arrows.

Fig. 3 illustrates the effect of BRB on expression of p21^WAF1^, γH2AX and ribosomal protein (rpS6) phosphorylated on Ser235/236 (rpS6^P^) in A549 cells, detected by phospho-specific Ab, induced to senescence by treatment with Mxt for 5 days. The Mxt-induced senescence of these cells manifests in dramatic increase in expression of p21 (34.5-fold), which is more pronounced than after 3 days of treatment with Mxt (Fig. 1). However, the increase in expression of p21 is markedly reduced in cells treated with Mxt in the presence of BRB and the reduction is BRB-dose dependent, starting with 56% at 5 μM and decreasing by 94% at 60 μM concentration. The induction of p21 by Mxt is paralleled by cell arrest in G_1_ and G_2_M phases of the cell cycle, and the arrest in G_2_M is to some extent reduced at 5 and 10 μM BRB (top panels, insets). The Mxt-induced senescence is also marked by 3.5-fold rise in expression of γH2AX and this rise is also attenuated, in a concentration-dependent fashion, by BRB, varying from 10% to 52% at 5 to 60 μM concentration of this isoquinoline. Phosphorylation of rpS6 on Ser235/236 is reduced by 11% in cells induced to senescence by Mxt alone. However, there is a dramatic further reduction in expression of this phosphorylated protein in cells growing in the presence of Mxt and BRB compared with Mxt alone, also in the dose-dependent mode, from 34% at 5 μM, to as much as 81% at 60 μM BRB concentration.

**Figure 4:**
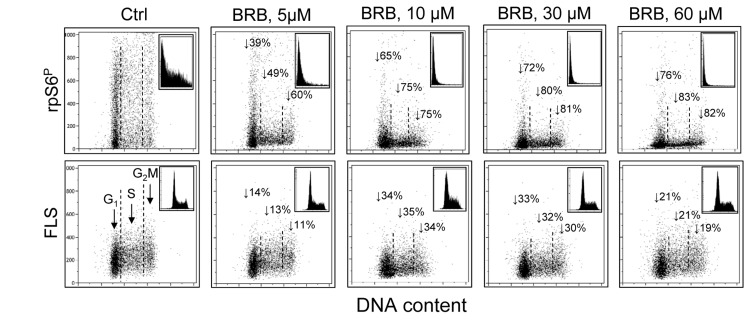
Suppression of rpS6 phosphorylation and reduction of size of human lymphoblastoid TK6 cells grown in the presence of BRB at 5 μM - 60 μM concentration Exponentially growing TK6 cells were untreated (Ctrl) or treated with BRB at concentrations as shown, for 24 h. Top panels: the bivariate distributions of rpS6^P^ expression versus DNA content. Figures show percent decrease in expression of the mean rpS6^P^ for cells at G_1_, S and G_2_M phases of the cell cycle, respectively, growing in the presence of BRB vis-à-vis to the untreated cells. Insets show the frequency histograms of rpS6^P^ expression for all cells in culture. Bottom panels: Bivariate distributions of cellular forward light scatter (FLS) versus DNA content. Percent decrease in of mean value of FLS, considered to represent cellular size [49,50], of G_1_, S and G_2_M of cells growing in the presence of BRB with respect to the untreated cells is shown with the arrows. Insets illustrate DNA content frequency histograms of cells from the respective cultures.

The results shown in Figs. 1-3 demonstrate that the induction of premature cellular senescence of A549 cell by treatment with Mxt is distinctly attenuated by BRB and the attenuation is already evident at its 5 μM concentration. In our prior study we observed that treatment of several cell types including A549, TK6, WI-38 cells and normal proliferating lymphocytes with different potential gero-suppressive agents reduced both, the mTOR- as well as DNA damage- signaling [33]. Among these agents was BRB which at 60 μM concentration was seen to markedly suppress the level of constitutive phosphorylation of mTOR, rpS6 and 4EBP1 as well as of H2AX. These findings were consistent with the notion that BRB had potential gero-suppressive properties combined with the ability protect DNA from endogenous oxidants [33]. In light of the current observation that 5-60 μM BRB suppresses induction of the premature senescence we have tested its ability at these lower concentrations to affect the level of constitutive mTOR signaling in cells not induced to premature senescence. Such low BRB concentrations are relevant in terms of the drug pharmacokinetics and its *in vivo* effects [45-48]. To this end we treated human lymphoblastoid TK6 cells, the cells which we explored in the prior study [33], with the range of BRB concentration as used for A549 cells. As is evident from the data in Fig. 4 growth of these cells in the presence of 5 – 60 μM BRB led to a distinct reduction of rpS6 phosphorylation. The reduction was evident already at 5 μM BRB and at that concentration its extent showed distinct cell cycle phase specificity. Namely at 5 μM the reduction was more pronounced in G_2_M- and S- phase cells, lowering expression of rpS6^P^ in these cells by 60% and 49%, respectively, compared with 39% for G_1_ cells. The degree of suppression of rpS6 phosphorylation was BRB-concentration dependent, reaching over 80% for the G_2_M and S-phase cells at its 60 μM concentration. The BRB concentration-dependency was quite apparent from the rpS6^P^ frequency histograms (Fig. 4 top panels, insets).

Interestingly, growth of TK6 cells in the presence of 5 - 60 μM of BRB for 24 h led to reduction of their size (Fig. 4, bottom panels). The reduction was estimated from analysis of the forward light scatter (FLS), which when measured by flow cytometry is considered to be a marker of cell size [49,50]. The diminished size of TK6 cells was evident already at 5 μM concentration of BRB and it was approximately of similar extent for cells in different phases of the cell cycle. The cell size reduction was more extensive at 10 μM and 30 μM than at 5 μM BRB concentration. No significant changes in the cell cycle distribution were seen at these BRB concentrations, as reflected by the DNA content frequency histograms (insets).

**Figure 5:**
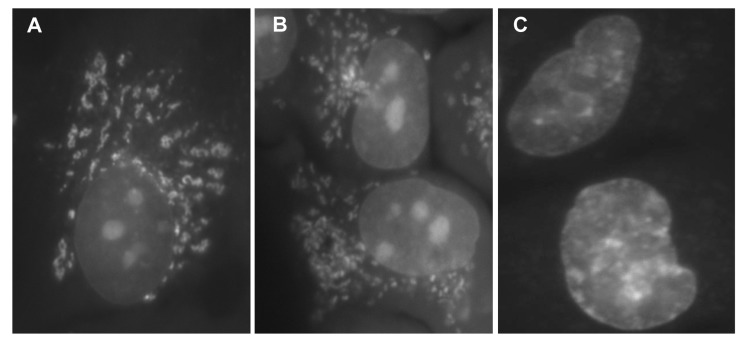
Detection of BRB fluorescence in live A549 cells exposed to this isoquinoline Exponentially growing A549 cells were treated with 5 μM BRB for 60 min, rinsed with PBS and examined under fluorescence microscopy. Immediately after illumination (the first min after exposure to UV) the BRB yellow fluorescence was localized almost exclusively in mitochondria (**A**). With extended time of illumination (2 min) intensity of fluorescence of mitochondria declined while nuclear and nucleolar fluorescence become more apparent (**B**). After 5 min exposure to UV no mitochondrial fluorescence was evident whereas intensity of nuclear and nucleolar fluorescence was distinctly increased (**C**). Images taken with the Nikon Microphot FXA; objective Fluor 40X.

**Figure 6:**
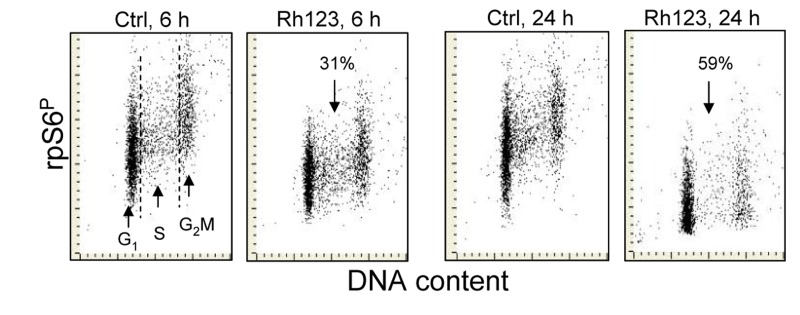
Effect of treatment of A549 cells with Rh123 on the level of expression of phosphorylated rpS6 Exponentially growing A549 cells were treated for 6 h or 24 h with 1 μM Rh123. Expression of rpS6^P^ in cytoplasm was detected by phosphospecific Ab and measured by the iCys laser scanning cytometry. The figures indicate percent decree in expression of RP-S6P in the Rh123 treated cells vis-à-vis the respective control (Ctrl) cells.

BRB is fluorescent and its fluorescence and localization in mitochondria in live cells was initially reported by Borodina and Zelenin in 1977 [51]. Its fluorescence is maximally induced by UV light at 421-431 nm and emission is within a wide range of green to yellow (514-555 nm) wavelength [52]. In binding to mitochondria BRB has an affinity to respiratory electron transport chain and the extent of its binding appears to correlate with mitochondrial potential ΔΨm [53-55]. This is in analogy to another mitochondrial probe rhodamine123 (Rh123) which is a widely used marker of mitochondrial energizing [56,57]. We have presently observed that 60 min exposure of A549 cells to BRB distinctly labels mitochondria (Fig. 6). This can be seen however for only for short period of time after exposure to blue light (< 3 min) or even shorter (< 1 min) to UV light. With longer exposures the BRB fluorescence disappears from mitochondria and undergoes translocation to nuclei and nucleoli (Fig. 5). It should be noted that BRB fluorescence was not apparent in cells that were fixed and subsequently rinsed, and thus it did not interfere with the subsequent measurement of fluorescent markers reporting mTOR/S6- and DNA damage- signaling.

The evidence in literature [53-55] and our observation (Fig. 5) indicate that in live cells BRB localizes in mitochondria where most likely it interferes with the electron transport chain. It may be suspected therefore that its presently measured effect namely attenuation of phosphorylation of rpS6 may be mediated by targeting electron transport in mitochondria. We have tested therefore the effect of a classical mitochondrial potential probe Rh123 [56,57] on rpS6 phosphorylation. As is evident from the data in Fig. 6 exposure of A549 cells in the presence of Rh123 leads to a decline in the level of constitutive phosphorylation of rpS6. The effect is not cell cycle-phase specific but related to time of exposure to Rh123, as 31% decrease is noted after 6 h and 59% after 24 h of treatment with Rh123, respectively.

## DISCUSSION

### Potential gero-suppressive properties of berberine

Several elements of stress-induced premature cellular senescence are relevant to aging. The key element, considered to be the major driving force of aging, is persistent activation of mTOR and its downstream target rpS6 kinase (p70S6 kinase; p70S6K), a serine/threonine kinase that phosphorylates rpS6. Phosphorylation of rpS6 induces protein synthesis at the ribosome [27,36,38]. When equilibrium between protein synthesis and its degradation (including by autophagy) is broken and synthesis prevails, cells become hypertrophic acquiring senescent (“growth imbalance”) phenotype, the phenomenon described nearly five decades ago [59]. There is an extensive and rapidly accumulating evidence in support of this mechanism as the primary inducer of premature cellular senescence as well as major contributor to organismal aging [20-28,59-63]. As mentioned in the Introduction we have tested seven different potential gero-suppressive agents in terms of their ability to attenuate the level of constitutive mTOR/S6 signaling in normal cell types and cell lines. Each of these agents was quite effective in causing the decline of this signaling [33]. Interestingly, they all also reduced the level of constitutive DNA damage response, considered to be a reporter of DNA damage by endogenous oxidants [30-32]. In continuation of these studies we initiated to assess whether these agents can modulate the induction of premature cellular senescence. The senescence was instigated by low level of persistent DNA damage maintained by cell exposure to DNA-targeting drugs mitoxantrone [39] or mitomycin C [40]. Such treatment was seen to induce replication stress manifesting by low level of DNA replication activity combined with the induction of DNA damage signaling viz. ATM activation and H2AX phosphorylation, and development of characteristic features of cellular senescence [39,40,64,65]. In the pilot experiments we observed that one of the investigated gero-suppressive agents, namely BRB, was particularly effective in attenuation of premature cell senescence. The present studies were designed to investigate this phenomenon in more detail, using BRB concentrations that are relevant to its potential pharmacological *in vivo* doses [45-48].

The present results clearly indicate that administration of BRB into cultures of A549 cells undergoing premature senescence reduced the development of senescent phenotype as revealed by analysis of cells morphometric features, activation of SA-β-gal and induction of CDK inhibitor p21^WAF1^. BRB also attenuated the level of mTOR/S6 signaling by lowering the level of phosphorylation of rpS6, as well as expression of γH2AX. All these effects were BRB-concentration-dependent and already evident at its lowest, 5 μM concentration. In parallel experiments, we observed that BRB also decreased rpS6 phosphorylation and reduced the size of TK6 cells (Fig. 4). In the prior study 60 μM BRB was seen to attenuate the level of constitutive mTOR/S6 and DNA damage signaling, and to reduce both the mitochondrial potential (ΔΨm) as well as the abundance of ROS [33]. Since as mentioned, the mechanisms of stress-induced cellular senescence and aging have much in common, collectively these data suggest that BRB can be effective at an *in vivo* achievable concentration [45-48,66,67] as a gero-suppressive agent.

The mechanism by which BRB exerts these effects may be through targeting mitochondria. Its localization in mitochondria was reported before [53-55] and presently shown in the case of A549 cells (Fig. 5). BRB in mitochondria is photolabile; even the short exposure to UV light results in a loss of its mitochondrial localization and apparent translocation into nuclei. The specific target appears to be the respiratory electron transport chain [53,54]; inhibition of the electron transport results in a decrease of ATP production which leads to an increase of AMP to ATP ratio which in turn triggers the AMP-activated protein kinase (AMPK) [68]. The inhibition of mTOR/S6 signaling, the event presently observed (Fig. 3), is one of the key effects of AMPK activation [27,28,36-38]. This mechanism is essentially identical to that induced by metformin, which also targets electron transport in complex 1 of mitochondria and in this way activates AMPK [69]. In fact, among its many clinical applications BRB, similar to metformin, has been promoted as an anti-diabetic supplement [70-72]. Since metformin was shown to extend lifespan of *C. elegans* [73,74] and even rodents [15,75,76] (although not of *Drosophila* [77]), it is reasonable to expect that BRB may demonstrate gero-suppressive properties as well.

In the present study we observed that exposure of A549 cells to the classic cationic mitochondrial probe Rh123, which also targets electron transport chain and monitors mitochondrial electrochemical potential ΔΨm [56,57], led to reduction of rpS6 phosphorylation (Fig. 6). It is thus possible that inhibition of the mitochondrial respiratory chain by other modalities, *via* similar mechanism of activation of AMPK and mTOR inhibition as in the case of metformin or BRB, may have potential gero-suppressive properties as well. It should be noted however, that these potential gero-suppressive agents may differ in other properties and may have different side effects. Thus, for example, whereas rapamycin extends lifespan of various organisms including vertebrates [16,78], it does not ameliorate some traits of animal aging [79,80]. The possibility of induction of autophagy as an additional mechanism counteracting cellular senescence and providing anti-aging benefits should be estimated in parallel with the inhibitory activity on the mTOR/S6 signaling [81-83]. The choice of an agent or perhaps a combination of several agents differing in primary binding site and/or mechanism of action that, would have maximal gero-suppressive properties and minimal side effects, has to be explored to assess the advantages of their use for attenuation of aging processes. Their analysis *in vitro* such as exploring potential in preventing the premature, stress-induced, cellular senescence as presently shown in the case of BRB, may offer an advantage over *in vivo* experiments testing animals' longevity, by yielding the data rapidly and economically.

BRB has a long history of medicinal use in both Ayurvedic and old Chinese medicine. More recently, BRB has found wide application to treat a variety of different maladies. However, because it has been used primarily as a dietary nutritional supplement the evidence of its clinical toxicity or side effects such as is generally being obtained from well monitored clinical trials is scarce but forthcoming [72,84]. Interestingly, many applications at which BRB was reported to have positive health effects relate to age-related diseases [84-89], including metabolic and cardiovascular risks [84], type 2 diabetes [45-47,70-72], atherosclerosis [68], senile osteoporosis [87], Alzheimer's disease [88], hypercholesterolemia [89] and diabetes-induced renal inflammation [90]. It is tempting to speculate that this diversity of medical benefits reportedly provided by BRB, having one common denominator namely organismal aging, stems from the gero-suppressive properties of this isoquinoline, as presently detected. It should be noted, however that BRB [91,92], similar as metformin [93-95], was shown to exert anticancer properties, suppressing growth and/or sensitizing cancer cells to various other anticancer modalities.

### *In vitro* assessment of gero-suppressive agents

Our prior studies [32-35,64] and the present results indicate that evaluation of effectiveness of potential anti-aging modalities can be achieved *in vitro* by monitoring their effect on mTOR/S6- and ROS-DNA damage signaling pathways that leads to attenuation of the stress-induced cellular senescence. Quantification of these effects is carried on using phospho-specific Abs that detect critical phosphorylation of the mTOR targets such as rpS6 and 4EBP1 concurrently with the level of ROS, ΔΨm, constitutive expression of γH2AX and activation of ATM, all measured in individual cells by multiparametric flow- or laser scanning- cytometry.

**Figure 7:**
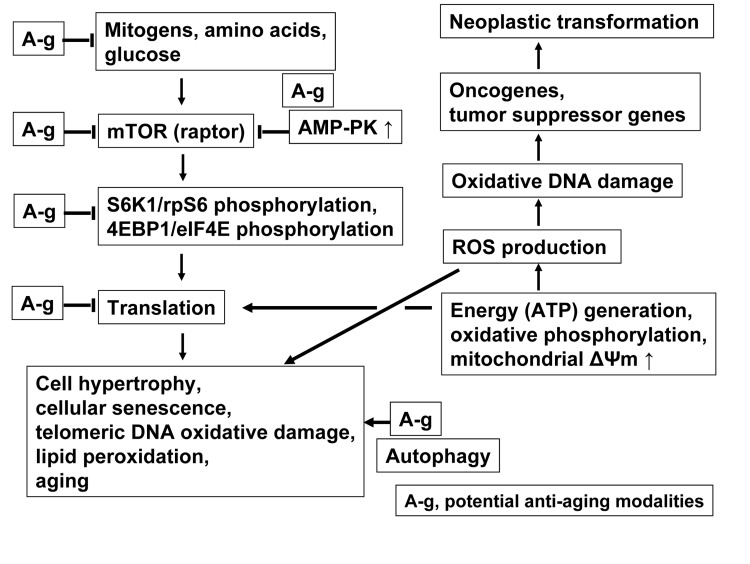
Schematic presentation of key pathways associated with cellular senescence and aging linking mTOR- and DNA damage- signaling The ongoing translation particularly during perturbed cell cycle progression (replication stress), is considered to be the major factor leading to senescence. Suppression of translation that may have anti-aging effect can be achieved at several steps along the mTOR signaling pathway (marked A-g), as discussed in the text. Activation of autophagy provides an additional gero-suppressive effect. The translation requires production of ATP and thus generates ROS that cause oxidative DNA damage, which when occurs at sites of oncogenes and tumor suppressor genes, may lead to neoplasia. The damage of telomeric DNA and lipid peroxidation by ROS further contributes to the senescent phenotype. The *in vitro* model of stress-induced cellular senescence as presently described can be used to evaluate potential gero-suppressive agents in terms of their effect in reduction of mTOR/S6- and DNA- damage signaling. See text for further details.

Fig. 7 presents key pathways associated with cellular senescence and aging linking mTOR/S6- and DNA damage- signaling, the targets of potential gero-suppressive agents that can be assessed this way. Reduction of signals activating m-TOR (raptor) pathway such as mitogens, growth factors and amino acids, triggering MAPK, Rsgs, MAP4K3, RaIA and hVps34, provide the outmost target for gero-suppression. These signals are suppressed by 2-deoxyglucose and other calorie restriction mimetics as well as by inhibitors of growth factors, primarily of IGF-1. Downstream of these signals, the mTOR activation is directly suppressed by its specific inhibitor rapamycin, and indirectly by activation of AMP-PK. Among effective activators of AMP-PK is metformin, as well as BRB. While the gero-suppressive effects of metformin are well documented [15,16] the present data demonstrate strongly suppressive effect of BRB on induction of the stress-induced premature cellular senescence. As mentioned, activation of AMP-PK can be achieved by targeting mitochondrial energy production, as it was shown with Rh123 (Fig. 6).

Since the increased rate of translation driven by rpS6 phosphorylation is the primary motor of cell growth in size (hypertrophy, “growth imbalance”, which is a characteristic phenotype of senescence) it is expected that inhibition of the translation rate through other means, downstream of mTOR activation, can also have gero-suppressive effects. For example, mimetics of amino acids that are not incorporated into protein but lower the rate of translation may have similar gero-suppressive effect. Likewise, reduction of ribosomal activity by such means as modification of the mRNA with the mRNA 5'-cap analogs [97, 98] or other approaches [99] may be gero-suppressive as well. Attenuation of the senescence phenotype that may be independent of translation is mediated by autophagy (Fig. 7) [100-102]. Specific inducers of autophagy, therefore, may have an additive effect with the mTOR inhibitors in terms of suppression of aging. However, most mTOR inhibitors are also activators of autophagy [100-102].

Our observation that the gero-suppressive agents inhibit constitutive level of mTOR activation concurrently with reduction of DNA damage signaling [33] provides evidence of a direct linkage between mTOR activation and oxidative DNA damage (Fig. 7). This is expected since translation requires production of energy (ATP) through oxidative phosphorylation which generates ROS; the increased ROS production (translation) leads to a greater oxidative DNA damage. It should be noted however that when ROS induce damage to telomeric sections of DNA this has an effect in promoting replicative senescence (Fig. 7). Likewise, the ROS-mediated lipid peroxidation is another marker of cellular senescence and aging.

Whereas mTOR/S6 signaling is the primary basis for induction of premature cell senescence and aging the endogenous ROS when cause DNA damage at sites coding for oncogenes or tumor suppressor genes predispose to neoplastic transformation (Fig. 7). Anti-oxidants (ROS scavengers) therefore are expected to be more effective in terms of chemo-prevention rather than as anti-aging modalities, and this indeed appears to be the case [103-106]. The attempts to attenuate aging processes including the increase in organismal longevity by antioxidants were largely unsuccessful [reviewed in 20]. On the other hand, most gero-suppressive agents were shown to have chemopreventive properties as well [106-112].

## MATERIALS AND METHODS

### Cells, Cell Treatment

*Human non-small cell lung carcinoma A549 cells*, obtained from American Type Culture Collection (ATCC; Manassas, VA), were grown in Ham's F-12K Nutrient Mixture (Mediatech, Inc., Manassas, VA) supplemented with 10% fetal bovine serum, 100 units/ml penicillin, 100 μg/ml streptomycin and 2 mM L-glutamine (GIBCO/BRL; Grand Island, NY) in 25 ml FALCON flasks (Becton Dickinson Co., Franklin Lakes, NJ) at 37.5 °C in an atmosphere of 95 % air and 5% CO_2_. The cells were maintained in exponential and asynchronous phase of growth by repeated trypsinization and reseeding prior to reaching sub-confluency. The cells were then trypsinized and seeded at about 1×10^4^ cells per chamber in 2-chambered Falcon Culture Slides (Beckton Dickinson). To induce cellular senescence A549 cells were treated with 2 nM mitoxantrone (Mxt; Sigma-Aldrich, St. Louis, MO) as described before [39]. Concurrently with Mxt berberine (BRB; Sigma-Aldrich) was included into cultures at a final concentration as shown in Figure legends. At the end of incubation medium from each chamber was aspirated, 1 ml of 1% methanol-free formaldehyde in phosphate buffered saline (PBS) was added and the cells fixed by gently rocking the slides at room temperature for 15 min. Following aspiration of the formaldehyde the chamber slides were disassembled and the slides submerged in 70% ethanol. The fixed slides were stored at 4°C before staining and analysis. *Human lymphoblastoid TK6 cells*, kindly provided by Dr. Howard Liber [96], were maintained in suspension in RPMI 1640 medium (GIBCO/Life Technologies) supplemented with L-glutamine (2 mM) and fetal bovine serum (10%), as described [33-35]. These cells were also exposed to 1 μM rhodamine 123 (Rh123; Sigma-Aldrich) to be assessed for expression of rpS6^P^ and forward light scatter by flow cytometry. Other details on cultures' treatment are presented in figure legends.

### Detection of γH2AX, rpS6^P^, p21^WAF1^ and activity of senescence-associated β-galactosidase

After fixation the cells were washed twice in PBS and with 0.1% Triton X-100 (Sigma-Aldrich) in PBS for 15 min and with a 1% (w/v) solution of bovine serum albumin (BSA; Sigma-Aldrich in PBS for 30 min to suppress nonspecific antibody (Ab) binding. The cells were then incubated in 1% BSA containing a 1:300 dilution of phospho-specific (Ser139) γH2AX mAb (Biolegend, San Diego, CA) and/or with a 1: 200 dilution of phosphospecific (Ser235/236), rpS6 Ab (Epitomics, Burlingame, CA) or 1;100 dilution of p21^WAF1^ Ab (Cell Signaling, Danvers, MA) at 4°C overnight. The secondary Ab was tagged either with AlexaFluor 488 or 647 fluorochrome (Invitrogen/Molecular Probes, used at 1:100 dilution in 1% BSA). The incubation was at room temperature for 45 min. Cellular DNA was counterstained with 2.8 μg/ml 4,6-diamidino-2-phenylindole (DAPI; Sigma-Aldrich) at room temperature for 15 minutes. Activity of senescence-associate β-galactosidase (SA-β-gal) was detected using the protocol provided by Chemicon's® Cellular Senescence Assay Kit (Millipore, Billerica, MA). After adding of Antifade (for fluorescence-labeled cells) or 50% glycerol in PBS (for (SA-β-gal) the slides were subjected to quantitative study by laser scanning cytometry. The protocol for measuring the chromatic (SA-β-gal) dye staining was provided by CompuCyte (Westwood, MA). The fixation, rinsing and labeling of A549 cells was carried out on slides, and TK6 cells in suspension. Other details have been described previously [64,112].

### Analysis of Cells by Cytometry

*A549 cells:* Cellular immunofluorescence representing the binding of the respective phospho-specific Abs as well as the blue emission of DAPI stained DNA was measured by laser scanning cytometer [39-41] iCys (CompuCyte) utilizing standard filter settings; fluorescence was excited with 488-nm argon, helium neon (633 nm) and violet (405 nm) lasers. Intensities of maximal pixel and integrated fluorescence were measured and recorded for each cell. At least 3,000 cells were measured per sample. Gating analysis was carried out as described in Figure legends. *TK6cells:* Intensity of cellular fluorescence was measured using a MoFlo XDP (Beckman-Coulter, Brea, CA) high speed flow cytometer/sorter. DAPI fluorescence was excited with the UV laser (355-nm), AlexaFluor 488, DCF and Rh123 with the argon ion (488-nm) laser. Although BRB is fluorescent [Fig. 5] it was not retained by the cells following their fixation and repeated washings and control experiments excluded the possibility that its fluorescence significantly contributed to analysis of the measured cells that could lead to a bias. Analysis of forward light scatter by flow cytometry provides information on cell size [49,50]. All experiments were repeated at least three times, representative data are presented. Other details of the particular experimental procedures were described before [64,112].
